# Evidence for positive allosteric modulation of cognitive-enhancing effects of nicotine in healthy human subjects

**DOI:** 10.1007/s00213-019-05363-4

**Published:** 2019-11-04

**Authors:** Britta Hahn, Megan E. Shrieves, Cory K. Olmstead, Marie B. Yuille, Joshua J. Chiappelli, Edna F. R. Pereira, Edson X. Albuquerque, William P. Fawcett

**Affiliations:** 1 Department of Psychiatry, Maryland Psychiatric Research Center, University of Maryland School of Medicine, P.O. Box 21247, Baltimore, MD 21228 USA; 2 Department of Epidemiology and Public Health, Division of Translational Toxicology, University of Maryland School of Medicine, 10 S. Pine St., Suite 900, Baltimore, MD 21201 USA

**Keywords:** Nicotine, Galantamine, Positive allosteric modulator, Cognition, Attention, SARAT, RVIP, CDT, Non-smokers

## Abstract

**Rationale:**

Cognitive benefits of nicotinic acetylcholine receptor (nAChR) agonists are well established but have generally been of small magnitude and uncertain clinical significance. A way of raising the effect size may be to facilitate agonist-induced responses by co-administering a nAChR positive allosteric modulator (PAM).

**Objective:**

The aim was to test whether galantamine, a PAM at several nAChR subtypes, can potentiate the cognitive-enhancing effects of nicotine.

**Methods:**

Twenty-six adult never-smokers were treated, in a double-blind counterbalanced sequence, with nicotine (7 mg/24 h, transdermally) and galantamine (4 mg, p.o.) combined, nicotine alone, galantamine alone, and double placebo. A low dose of galantamine was chosen to minimize acetylcholinesterase inhibition, which was verified in blood assays. In each condition, participants were tested with three cognitive tasks.

**Results:**

Nicotine significantly improved reaction time (RT) and signal detection in a visuospatial attention task and the Rapid Visual Information Processing Task. Galantamine did not modulate these effects. A trend toward RT reduction by galantamine correlated with acetylcholinesterase inhibition. In a change detection task, there were no effects of nicotine or galantamine alone on accuracy or RT. However, both drugs combined acted synergistically to reduce RT. This effect was not associated with acetylcholinesterase inhibition.

**Conclusions:**

A pattern consistent with allosteric potentiation of nicotine effects by galantamine was observed on one of six performance measures. This may reflect specific nAChR subtype involvement, or additional pharmacological actions of galantamine may have overshadowed similar interactions on other measures. The finding suggests that allosteric potentiation of nAChR agonist-induced cognitive benefits is possible in principle.

## Introduction

Several disease states marked by cognitive deficits, most prominently schizophrenia and Alzheimer’s disease, involve nicotinic acetylcholine receptor (nAChR) hypofunction (Adams and Stevens 2007; Hong et al. 2011; Kendziorra et al. 2011; Perry et al. 2000; Petrovsky et al. 2010; Wing et al. 2012) and may benefit from treatments that enhance nAChR activity (Levin and Rezvani 2002; Singh et al. 2004). Acute cognitive benefits of the prototypical non-selective nAChR agonist nicotine are well established, particularly on attention but also on sensory information processing and mnemonic processes (Hahn 2015; Heishman et al. 2010; Newhouse et al. 2011), although the clinical benefit of chronic treatment with nicotine is unclear. Drug development efforts have been invested into subtype-selective nAChR agonists for the above conditions. Effects with both α4β2- and α7-selective nAChR agonists have generally been in the expected direction, but tend to be of small magnitude and uncertain clinical significance (Haydar and Dunlop 2010; Radek et al. 2010; Wallace et al. 2011). Many compounds failed clinical trials due to limited efficacy (Haydar and Dunlop 2010; Hurst et al. 2013).

A way of raising the effect size ceiling may be to co-administer a nAChR agonist and a nAChR positive allosteric modulator (PAM). PAMs do not activate nAChRs on their own. Instead, PAMs bind to an allosteric site on the receptor and facilitate agonist-induced responses (Williams et al. 2011). Some, although not all (Gronlien et al. 2007), PAMs reverse desensitization of a fraction of nAChRs, specifically in the presence of low to intermediate agonist concentrations (Williams et al. 2011). Thus, through partial reversal of desensitization or other mechanisms, combined PAM and low-dose agonist treatment may enhance nAChR activity and associated behavioral effects to a greater degree than agonist treatment alone. Despite much discussion and interest in nAChR PAMs, the ability of a PAM to enhance cognitive effects of nicotine or other nAChR agonists has never been tested in a systematic manner in either animals or humans. Dual administration studies performed in people with schizophrenia (Choueiry et al. 2019; Deutsch et al. 2013) were not designed to differentiate between the effects of the PAM, the agonist, or their combination.

To date, the only nAChR PAM commercially available for human use is the acetylcholinesterase (AChE) inhibitor galantamine, approved by the Food and Drug Administration for the treatment of mild to moderate Alzheimer’s disease. Galantamine is a nAChR PAM at concentrations found in the human brain after clinical doses (Coyle et al. 2007; Villarroya et al. 2007). It has been shown to potentiate α4β2, α3*, α6β4, and α7 nAChR currents induced by acetylcholine, nicotine, or epibatidine, causing long-lasting increases in nAChR response amplitude and frequency (Dajas-Bailador et al. 2003; Samochocki et al. 2003; Santos et al. 2002). Thus, while galantamine’s AChE inhibitory action increases concentrations of acetylcholine in the synaptic cleft, its nAChR PAM action renders the nAChR more responsive to acetylcholine and exogenous nAChR agonists (Albuquerque et al. 2009). Importantly, the concentration range for galantamine’s PAM action appears to be slightly below that for AChE inhibition (Coyle et al. 2007). Thus, it appears that a bias toward its PAM action can be achieved by testing a small dose of galantamine and minimizing AChE inhibition.

The aim of the present study was to test whether performance-enhancing effects of a nAChR agonist can be potentiated by the co-administration of a nAChR PAM. For this proof-of-principle study, we tested the interaction of a low dose of galantamine with the prototypical nAChR agonist nicotine on a broad array of cognitive functions in healthy non-smoker. Non-smokers were selected to avoid potential confounds related to chronic nicotine exposure, such as neuroadaptive changes and nicotine withdrawal. Even at small doses, galantamine may have additional pharmacological actions; however, any potentiation of nicotine effects could not be explained by AChE inhibition but by allosteric potentiation of nAChR activity. Our specific predictions were no or little effect of galantamine alone, but larger performance-enhancing effects of nicotine in the presence of galantamine than in its absence.

## Methods

### Participants

Out of 43 healthy non-smokers enrolled in the study, 27 completed it (16 females, 11 males; 10 African American, 2 Asian, 13 Caucasian, 2 Hispanic). Reasons for non-completion were adverse effects in 8 cases (7 cases of vomiting; 1 case of nausea, jitteriness, and palpitations), no longer meeting inclusion criteria in 2 cases, and withdrawal for personal reasons in 6 cases. Non-completers were replaced; our target was to have at least 24 completers based on power calculation indicating that an interaction of medium effect size could be detected with this sample size. One study completer was excluded from analyses of performance data because this subject’s performance in two of the three tasks was marked by a large proportion of no-response trials, suggesting a lack of task engagement.

The remaining 26 subjects who completed the study were 22–51 years of age (mean ± SD: 33.4 ± 10.4) with 13–22 years of education (16.3 ± 2.3). Participants were recruited from the local community through internet advertising, flyers, and referrals, and gave written informed consent for a protocol approved by the University of Maryland Baltimore Institutional Review Board. Participants had no more than 40 cigarettes in their lifetime and no nicotine exposure in the last year. Use of centrally active medications, pregnancy, history of neurological or psychiatric disorders including drug abuse, significant liver or kidney impairment, heart problems, hyper- or hypotension, and learning disability were exclusion criteria.

### Drugs

Nicotine patches were over-the-counter Nicoderm CQ patches (GlaxoSmithKline, Brentford, Middlesex, UK) releasing 7 mg of nicotine in 24 h, close to the lowest dose available. Placebo patches were generated using AquaHeal Hydrogel Bandages (Spenco Medical Corporation), cut to size and with identifying labeling removed. The hydrogel bandages closely resemble the nicotine patch in color and consistency. The nicotine or size-matched placebo patch was placed on the inside of an adhesive bandage on the day of the study and sealed in a small ziplock bag until application. The adhesive bandage with patch was applied by a study nurse not involved in any other study procedures.

Galantamine HBr immediate release tablets (Patriot Pharmaceuticals, Horsham, PA) were ground up and packaged into capsules for p.o. administration at a dose of 4 mg per capsule by an in-house compounding pharmacist, who also produced matching placebo capsules containing microcrystalline cellulose. The low dose of galantamine was chosen to induce a bias toward its PAM action (Coyle et al. 2007).

### Study design and procedures

**Fig. 1:**
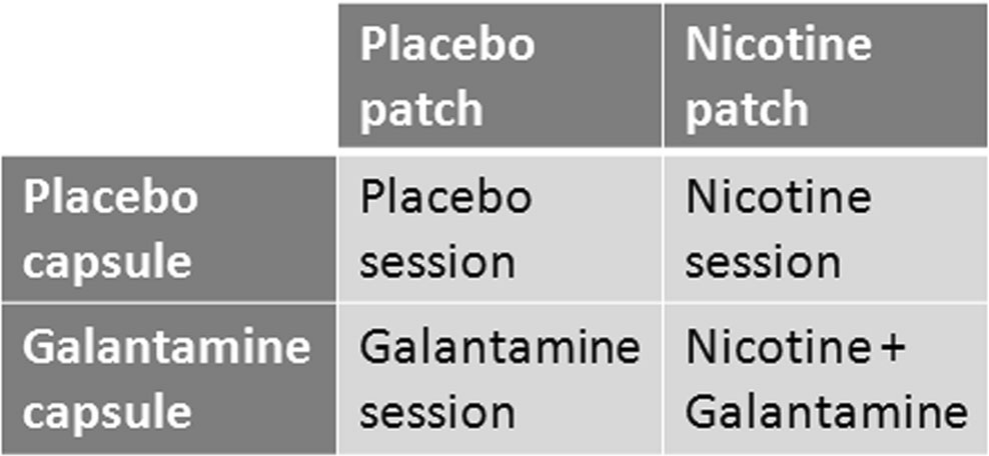
Experimental design

The study adopted a double-blind within-subject design. Each participant was tested on four separate days. On each day, a skin patch was applied, and a capsule was administered. On one day, both the patch and the capsules were a placebo (placebo session). On another day, the patch was a nicotine patch (7 mg/24 h) and the capsule was a placebo (nicotine session); on another, the patch was a placebo and the capsule contained 4 mg of galantamine (galantamine session); and on another day, the patch was a nicotine patch (7 mg/24 h) and the capsule contained 4 mg of galantamine (nicotine + galantamine session). Thus, the four conditions followed a 2 × 2 factorial design (see Fig. 1) and were tested in a sequence that was counterbalanced across participants to the degree possible.

The study involved six total visits: one consent and screening visit, one training visit, and the four test sessions, the latter scheduled at least 1 week apart to ensure complete drug washout and normalization of any potential secondary drug effects between sessions. Screening included a medical history and physical exam, an electrocardiogram, blood and urine labs, a vision test, and tests for drug use, smoking, and pregnancy. During the training visit, participants were given task instructions and performed a full-length version of each of the cognitive tasks described below, to minimize practice effects between test sessions.

Each test session took approximately 7 h. Upon arrival in the morning, participants were tested for fever and recent alcohol use or smoking, and a urine sample was tested for pregnancy and drug use, all of which had to be negative for the session to proceed. Resting blood pressure and heart rate measurements were taken, and participants completed a side effect checklist, rating possible side effects of nicotine and galantamine (restlessness, weakness/fatigue, dizziness, headache, dry mouth, nausea, abdominal pain, sweating, palpitations, jitteriness, sleepiness, diarrhea, decreased appetite, stomach discomfort, difficulty urinating) as none (1), mild (2), moderate (3), or severe (4). Participants then completed the Profile of Mood States (POMS), an adjective rating questionnaire considered a standardized subjective mood state inventory (McNair et al. 1971).

Next, the study patch was administered. Vital signs and the side effect checklist were obtained hourly thereafter. During the drug absorption period, participants were permitted to read, watch movies, or use the internet. Three and a half hours after patch administration, participants swallowed the study capsule, after which vital signs and the side effect checklist were obtained every 30 min. Five hours after patch application (1.5 h after capsule administration), the POMS was again completed, and cognitive testing began. This timing was based on peak drug concentrations after administration, as nicotine plasma concentrations have been shown to reach asymptote by 5 h post-patch administration (Fant et al. 2000; Gupta et al. 1993), and galantamine plasma concentrations reach *t*_max_ 1.2–1.6 h after p.o. administration of a 4-mg dose (Zhao et al. 2002). The order of the cognitive tasks always remained the same: first the Spatial Attentional Resource Allocation Task, then the Rapid Visual Information Processing Task, and last the Change Detection Task. Testing took approximately 1.5 h in total. Vital signs were measured after the first task. Immediately after cognitive testing, the POMS and side effect checklist were completed and vital signs were taken one last time, and a 5-ml blood sample was obtained from a forearm vein for analysis of nicotine concentrations and AChE activity. The blood draw was performed approximately 3:15 h after galantamine dosing.

### Equipment

All tasks were performed on a 19-in. 5:4 IPS LCD monitor with a screen resolution of 1280 × 1024 and a refresh rate of 60 Hz. Responses were recorded using a Logitech F310 gamepad controller. Only the left and right bumper buttons were used. In tasks involving a single button, subjects responded with their dominant hand. All tasks were created and run in E-Prime version 2.0.

### Task paradigms

#### Spatial Attentional Resource Allocation Task

**Fig. 2:**
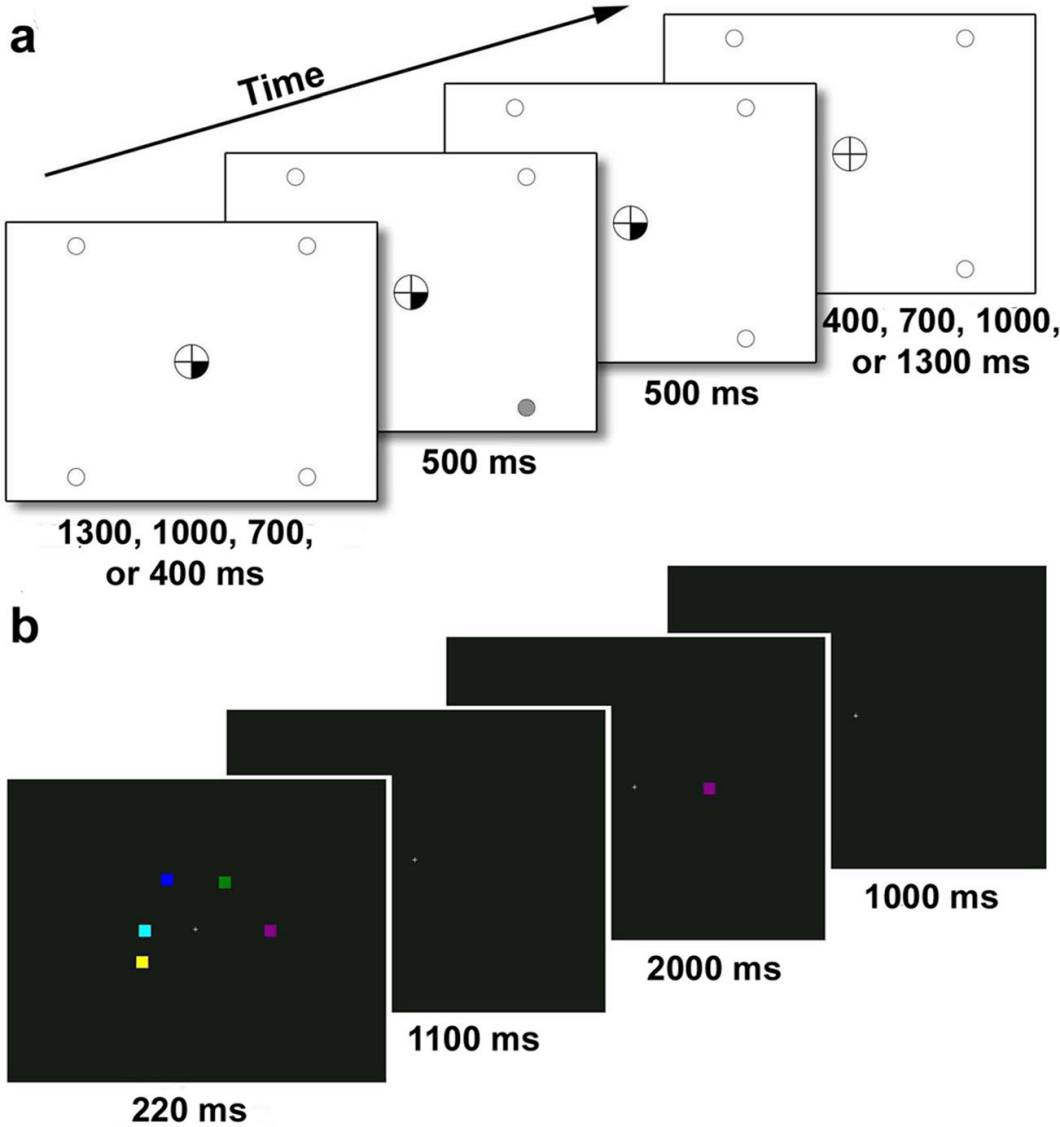
Components of a trial in the Spatial Attentional Resource Allocation Task (**a**) and the Change Detection Task (**b**)

The Spatial Attentional Resource Allocation Task (SARAT) is a visuospatial stimulus detection paradigm (Hahn et al. 2006), shown to be sensitive to the performance-enhancing effects of nicotine (Hahn et al. 2013; Hahn et al. 2007). Participants fixated on a quartered circle in the center of the screen (diameter approximately 2.6° of visual angle), black against a light gray (10% contrast; 130 cd/m^2^) background (Fig. 2a). They were instructed to respond as quickly as possible when detecting a 500-ms target stimulus appearing in one of four locations in the corners of the screen, marked by circular place holders (diameter 1.3° of visual angle), positioned at 10° of visual angle.

A cue in the central circle preceded the target signal by 400, 700, 1000, or 1300 ms, chosen randomly to make target onset temporally unpredictable and discourage anticipatory responding to the cue. Either one quarter turned black to indicate the location of the upcoming target (predictive cue), or all 4 quarters turned black (non-predictive cue). Predictive cue trials allowed for a narrow attentional focus, while non-predictive cue trials required attention to be spread widely to encompass the entire display. Participants were asked to respond as quickly as possible with their dominant index finger upon detecting a target. Targets were presented in the continued presence of the cue and consisted of peripheral placeholders filling with a gray (40% contrast) and white checkerboard pattern of 3 × 3 pixels each. The cue persisted for 500 ms after target offset. Only task background was then displayed for an inter-trial interval (ITI) of 400, 700, 1000, or 1300 ms.

The task was presented in eight 5-min blocks of 60 trials each: 30 predictive cue trials, of which six had no target to discourage anticipatory responding to the cue, and 30 non-predictive cue trials, of which six had no target. To increase the temporal jitter of the task and augment stimulus detection demands, 30 additional 2.7-s periods during which only task background was presented were interspersed randomly between trials. The entire task took approximately 45 min to complete.

#### Rapid Visual Information Processing Task

The Rapid Visual Information Processing Task (RVIPT) requires the maintenance of intense rapid information processing and working memory demands over time. Performance therefore reflects processing speed, sustained attention, and working memory.

The RVIPT has been used extensively to evaluate the cognitive effects of cholinergic agents and is sensitive to the performance-enhancing effects of nicotine (Foulds et al. 1996; Warburton and Mancuso 1998; Wesnes and Warburton 1984). The task consists of a string of digits (1 through 9), presented one at a time at a rate of 100/min. Each digit was presented for 600 ms, with no ITI. Participants were instructed to respond with their dominant index finger when they identified three consecutive odd or even digits. Responses within an 1800-ms time window following the onset of the last digit of a target sequence were considered hits; all other responses were considered false alarms. On average, 8 target sequences were presented per minute. The number of digits separating targets ranged from 4 to 29. The task was performed in two blocks of 15 min each, with a break between to ensure feasibility.

#### Change Detection Task

The Change Detection Task (CDT) is a visual short-term memory task (Luck and Vogel 1997) and was included as a probe for potential mnemonic drug effects. It is relatively inconducive to verbal rehearsal. A 220-ms encoding array of either 1 or 5 colored squares was presented (Fig. 2b). Possible colors were red, magenta, purple, yellow, white, blue, cyan, green, olive, and teal. Half of the trials showed 5 colored squares and the other half showed 1 colored square. After a 1100-ms retention interval, one square reappeared for 2000 ms, and participants determined whether this square was of the same or a different color than the square previously displayed at this location. On half the trials, the color was the same, and on the other half, the color changed. Participants responded “same” with a right button press, and “different” with a left button press. Trials were separated by a 1000-ms inter-trial interval. The task consisted of 180 total trials, presented over 5 blocks of 36 trials each, with short breaks between blocks. Total task duration was 13 min.

### Blood analyses

Immediately after the blood draw at the end of each test session, 0.2 ml of whole blood was pipetted off, and the rest of the sample was centrifuged to separate plasma from red blood cells. Whole blood and plasma samples were frozen at − 80 °C until analysis upon study completion. Only samples from study completers were analyzed.

Analyses of nicotine and cotinine plasma concentrations were performed by NMS Labs (Willow Grove, PA) by high-performance liquid chromatography/tandem mass spectrometry (LC-MS/MS). Because smoking abstinence was considered sufficiently verified, only samples from the nicotine session and nicotine + galantamine session were analyzed. The reporting limits were 2.5 ng/ml for nicotine and 5 ng/ml for cotinine.

Whole blood AChE activity was determined using a modification of the radiometric cholinesterase procedure (Johnson and Russell 1975). In brief, individual whole blood samples (45 μl) pretreated with the selective butyrylcholinesterase inhibitor tetraisopropyl pyrophosphoramide (100 μM) were incubated with acetylcholine (5 μl, 0.1 M ACh [acetyl-3H (20 μCi/ml)]) at room temperature for 3 min. The reaction was stopped with a 100-μl aqueous solution of chloroacetic acid (0.50 M), sodium chloride (1 M), and sodium hydroxide (0.25 M), and samples were clarified by centrifugation. The clarified samples (140 μl) were transferred to a scintillation fluor cocktail consisting of 90% (v/v) toluene, 10% (v/v) 3-metyl-1-butanol, 0.03% (w/v) 1,4-bis(5-phenyl-2-oxazolyl), and 0.05% (w/v) 2,5-diphenyloxazole (final reaction volume = 4 ml). The mix was vortexed for 60 s. The amount of tritiated acetate in the organic phase was measured by liquid scintillation counting for 2 min (Tri-Carb 2900TR, Perkin Elmer). Each sample was assayed in triplicate and counts were corrected for background by subtraction of counts obtained for whole blood samples devoid of cholinesterase activity.

### Statistical analysis

Vital signs and each subjective state scale from the side effect checklist were analyzed by three-factor ANOVA with nicotine, galantamine, and time as within-subject factors, including only the last four measurement time points at which both nicotine and galantamine absorption had taken place (if administered).

Each of the seven POMS scales (tension/anxiety, depression, anger/hostility, vigor/activity, fatigue, confusion, total mood disturbance) was analyzed by three-factor ANOVA with nicotine, galantamine, and time (baseline, pre-test, post-test) as within-subject factors.

Nicotine and cotinine plasma concentrations were compared between the nicotine session and the nicotine + galantamine session by paired samples *t* tests. AChE activity was compared between the four drug conditions by two-factor ANOVA for repeated measures with nicotine (present vs. absent) and galantamine (present vs. absent) as within-subject factors.

#### SARAT

Average RT and percentage of omission errors were analyzed by separate three-factor ANOVAs with nicotine, galantamine, and cue type (predictive vs. non-predictive) as within-subject factors.

#### RVIPT

Because of the large number of opportunities to make a false alarm, the false alarm rate was < 1% for all but one participant. Consequently, the sensitivity index A’ (Grier 1971) yielded a virtually identical performance pattern across conditions as the hit rate. Expectably, analysis of the false alarm rate did not yield any significant effects; thus, analyses reported here focus on the hit rate (percentage of target detections out of all targets presented) and mean RT. These variables were analyzed by three-factor ANOVA with nicotine, galantamine, and time period (3 periods of 10 min each) as within-subject factors.

#### CDT

Trials without a response (mean ± SD 1.2 ± 2.9 trials) were excluded from analyses. Accuracy (percentage of correct responses out of all response trials) and mean RT were analyzed by separate three-factor ANOVAs with nicotine, galantamine, and set size (1 vs. 5) as within-subject factors.

Nicotine × galantamine interactions on any performance measure, which were of primary interest in this study, were Bonferroni-corrected for six analyzed performance measures. Any performance effects of galantamine underwent Pearson’s correlation with AChE inhibition by galantamine. Effect sizes are reported as partial eta squared (*η*_*p*_^2^), with *η*_*p*_^2^ = 0.06 generally considered a medium and *η*_*p*_^2^ > 0.14 a large effect size (Cohen 1988). Significance testing was based on *P* < 0.05, two-sided.

## Results

### Adverse effects by drug conditions

Out of the 8 participants who were excluded due to greater-than-mild side effects (see above), 5 experienced side effects in the nicotine session and 3 in the nicotine + galantamine session. Two of the latter 3 participants dropped out before the capsule was administered. Thus, side effects were largely, if not entirely, related to nicotine administration. The one participant who got sick after combined nicotine and galantamine administration had previously completed the nicotine session without side effects, suggesting that galantamine may have potentiated adverse effects of nicotine in this case. However, one participant who experienced side effects in the nicotine session had previously completed the nicotine + galantamine session without side effects, suggesting that day-to-day variation in other factors influenced the response to nicotine.

**Fig. 3:**
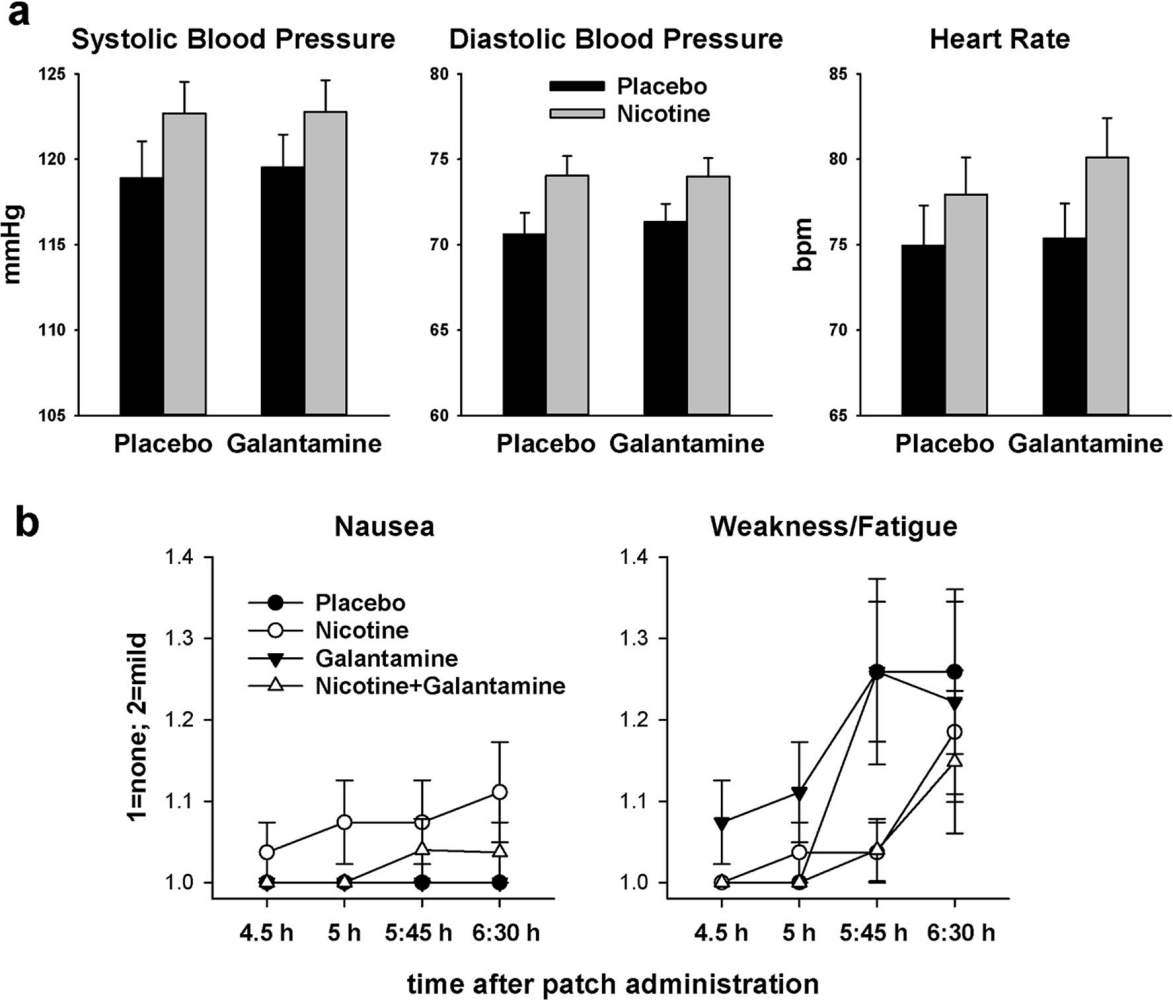
Effects of nicotine and galantamine on vital signs averaged over the last four measurement time points (**a**) and on self-report scales from the side effect checklist (**b**). Error bars reflect SEMs. Possible ratings on the self-report scales are 1 = none, 2 = mild, 3 = moderate, and 4 = severe

In the 27 completers, vital signs and the subjective state variables of the side effect checklist were analyzed by three-factor ANOVA (nicotine × galantamine × time) including the last four measurement time points: 4.5 h after patch administration (= 1 h after capsule administration); 5 h post-patch (= 1.5 h post-capsule, start of testing); 5:45 h post-patch (= 2:15 h post-capsule, mid-testing); and 6:30 h post-patch (= 3 h post-capsule, post-testing). There were no main effects or interactions involving galantamine. However, there was a significant main effect of nicotine on systolic blood pressure, diastolic blood pressure, and heart rate [*F*(1,26) > 17.6, *P* < 0.001 in each case] reflecting increases in the presence of nicotine (Fig. 3a). There were also main effects of nicotine on nausea [*F*(1,26) = 5.90, *P* = 0.022] and weakness/fatigue [*F*(1,26) = 4.42, *P* = 0.045]. Six participants reported nausea (all “mild”) at at least one of the time points analyzed: 4 in the nicotine session and 2 in the nicotine + galantamine session. The effects of nicotine on weakness/fatigue interacted with time [*F*(1,26) = 3.64, *P* = 0.016]: weakness/fatigue increased with cognitive testing, and nicotine appeared to alleviate this increase (Fig. 3b).

### Subjective drug effects as measured by the POMS

There were no significant main effects of nicotine or galantamine on any of the seven POMS subscales, and no nicotine × galantamine interactions.

### Nicotine and cotinine concentrations in blood plasma

For one participant, we were unable to obtain a blood sample in the nicotine session. In the other subjects, nicotine concentrations averaged 6.04 ± 1.45 ng/ml (range 2.8–9.6) in the nicotine session and 6.20 ± 1.71 ng/ml (range 2.7–9.2) in the nicotine + galantamine session [*t*(24) = 0.47, *P* = 0.65], comparable to the 5.9 ng/ml average plasma concentration observed with a nicotine patch of the same dose in smokers (Gorsline et al. 1993). Cotinine concentrations averaged 30.7 ± 9.7 ng/ml in the nicotine session and 33.8 ± 11.5 ng/ml in the nicotine + galantamine session [*t*(24) = 1.54, *P* = 0.14].

### Effects of galantamine on AChE activity in whole blood

**Fig. 4:**
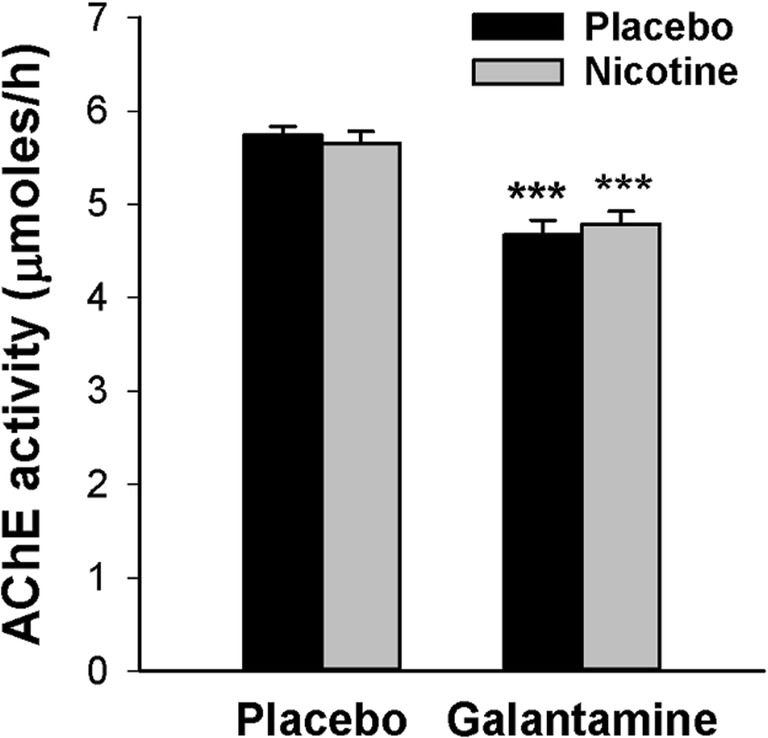
Average (± SEM) acetylcholinesterase (AChE) activity in whole blood in each drug condition. ***P < 0.001 in paired *t* tests comparing the galantamine session to the placebo session and the nicotine + galantamine session to the nicotine session

A low dose of galantamine (4 mg) was chosen with the purpose of minimizing AChE inhibition and associated behavioral effects. Figure 4 shows mean AChE activity in whole blood samples obtained at the end of each test session. AChE inhibition by galantamine was modest (16.8%) but statistically significant [main effect of galantamine: *F*(1,24) = 101.0, *P* < 0.001] and consistent with the 15% reduction in AChE activity reported by a previous study employing the same dose of galantamine (Morasch et al. 2015). There was no main effect of nicotine [*F*(1,24) = 0.00, *P* = 0.99] and no galantamine × nicotine interaction [*F*(1,24) = 1.25, *P* = 0.27].

### Drug effects on task performance

#### SARAT

**Fig. 5:**
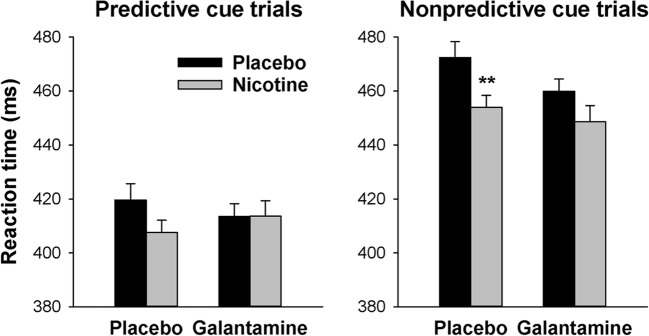
Effects of nicotine and galantamine on reaction time in the Spatial Attentional Resource Allocation Task. Bars reflect the mean performance in each drug condition. Error bars reflect SEMs, adjusted to remove between-subject variability in the average performance across dose levels (Cousineau 2007; Morey 2008) to yield variability related to interindividual differences in drug effect. **P < 0.01 in paired *t* test comparing performance after nicotine to performance after vehicle

Responses were slower and there were more omission errors for non-predictive than predictive cue trials [main effect of cue type on RT: *F*(1,25) = 66.8, *P* < 0.001, *η*_*p*_^2^ = 0.73; omissions: *F*(1,25) = 4.84, *P* < 0.037, *η*_*p*_^2^ = 0.16]. Nicotine shortened RT [*F*(1,25) = 5.40, *P* = 0.028, *η*_*p*_^2^ = 0.18] and reduced omission errors overall [*F*(1,25) = 5.78, *P* = 0.024, *η*_*p*_^2^ = 0.19]. There were no main effects of galantamine [RT: *F*(1,25) = 0.54, *P* = 0.47, *η*_*p*_^2^ = 0.02; omissions: *F*(1,25) = 0.07, *P* = 0.80, *η*_*p*_^2^ = 0.003] and no nicotine × galantamine interactions [RT: *F*(1,25) = 1.22, *P* = 0.28, *η*_*p*_^2^ = 0.05; omissions: *F*(1,25) = 1.20, *P* = 0.29, *η*_*p*_^2^ = 0.05]. However, on RT, the effects of cue type interacted with both nicotine [*F*(1,25) = 11.3, *P* = 0.002, *η*_*p*_^2^ = 0.31] and galantamine [*F*(1,25) = 11.5, *P* = 0.002, *η*_*p*_^2^ = 0.31]. Figure 5 suggests that both drugs reduced RT in non-predictive more than in predictive cue trials. Paired *t* tests comparing nicotine and placebo collapsed over levels of galantamine confirmed that nicotine reduced RT in non-predictive cue trials [*t*(25) = 3.17, *P* = 0.004, *η*_*p*_^2^ = 0.29; *P* = 0.016 after Bonferroni correction for 4 comparisons] but not in predictive cue trials [*t*(25) = 1.23, *P* = 0.21, *η*_*p*_^2^ = 0.06]. *t* tests comparing galantamine and placebo collapsed over levels of nicotine found no significant effect in either predictive [*t*(25) = 0.004, *P* > 0.99, *η*_*p*_^2^ = 0.00] or non-predictive cue trials [*t*(25) = 1.42, *P* = 0.17, *η*_*p*_^2^ = 0.07].

**Fig. 6:**
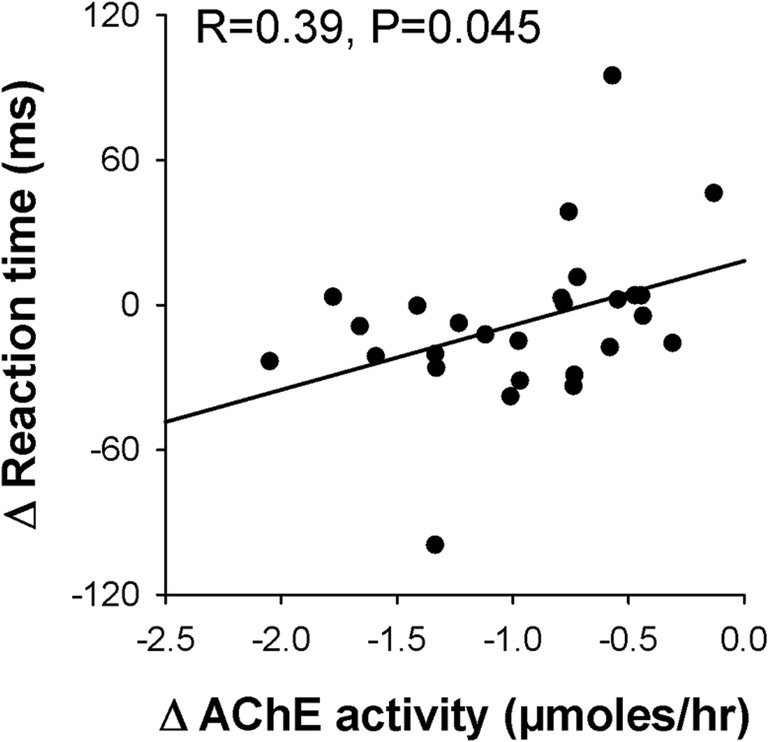
Correlation between the effect of galantamine on AChE activity and on SARAT reaction time across cue types. For both variables, difference (Δ) values were derived by subtracting the average value across the two no-galantamine sessions (placebo session and nicotine session) from the average value across the two sessions involving galantamine administration (galantamine session and nicotine + galantamine session)

To test whether the trend for galantamine to reduce RT in non-predictive cue trials reflected residual AChE inhibition, individual participants’ RT and AChE activity, averaged over non-galantamine test days (i.e., the placebo and nicotine sessions), was subtracted from values averaged over galantamine test days (the galantamine and nicotine + galantamine sessions) and underwent Pearson’s correlation. There was a significant correlation between the effects of galantamine on AChE activity and on RT (*R* = 0.39, *P* = 0.045), reflecting greater RT reduction in participants with greater AChE inhibition (Fig. 6).

#### RVIPT

**Fig. 7:**
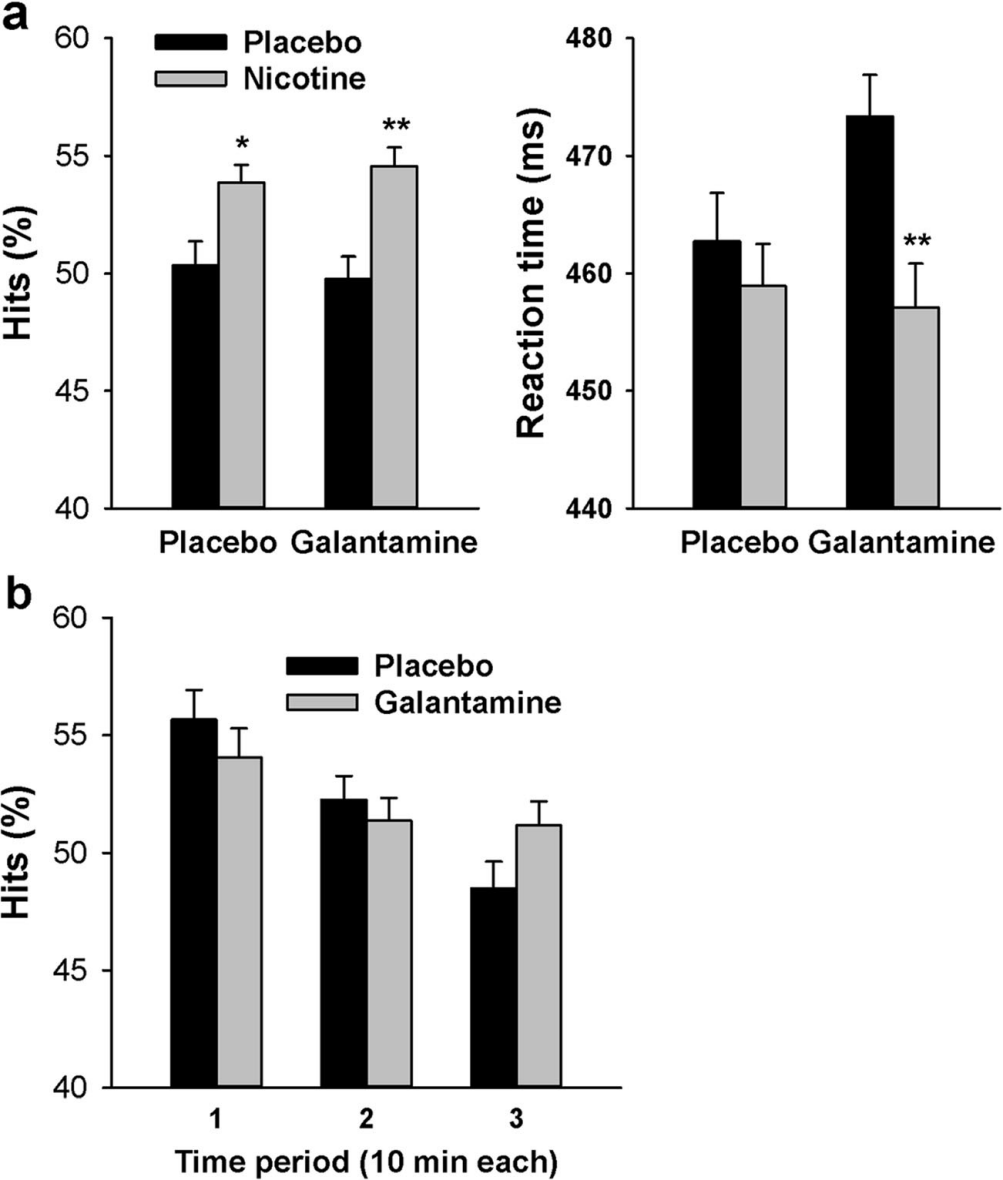
**a** Effects of nicotine and galantamine on hit rate and reaction time in the Rapid Visual Information Processing Task. Bars reflect the mean performance in each drug condition. *P < 0.05, **P < 0.01 in paired *t* tests comparing performance after nicotine to performance after vehicle. **b** Effects of galantamine, averaged over the presence and absence of nicotine, on hit rate in each of three 10-min time periods. Error bars in **a** and **b** reflect SEMs, adjusted to remove between-subject variability in the average performance across dose levels (Cousineau 2007; Morey 2008)

Figure 7a shows that nicotine increased hit rate [main effect: *F*(1,25) = 13.1, *P* < 0.001, *η*_*p*_^2^ = 0.34] and reduced RT [*F*(1,25) = 6.94, *P* = 0.014, *η*_*p*_^2^ = 0.14]. There was no significant main effect of galantamine on hit rate [*F*(1,25) = 0.004, *P* = 0.95, *η*_*p*_^2^ = 0.00] or RT [*F*(1,25) = 0.000, *P* > 0.99, *η*_*p*_^2^ = 0.00]. The effects of nicotine appeared larger in the presence of galantamine, in part because galantamine alone appeared to slow RT; however, the nicotine × galantamine interaction was not significant on RT [*F*(1,25) = 2.27, *P* = 0.15, *η*_*p*_^2^ = 0.08] or hit rate [*F*(1,25) = 0.53, *P* = 0.47, *η*_*p*_^2^ = 0.02]. There was a significant main effect of time period on both hit rate [*F*(1,25) = 12.4, *P* < 0.001; *η*_*p*_^2^ = 0.33] and RT [*F*(1,25) = 3.52, *P* = 0.037; *η*_*p*_^2^ = 0.12], reflecting performance decrement with time on task in both cases. The only significant interaction involving time period was with galantamine on hit rate [*F*(1,25) = 3.43, *P* = 0.04; *η*_*p*_^2^ = 0.12; all other *P* > 0.3], which was based on galantamine attenuating the decrement over time (Fig. 7b).

#### CDT

**Fig. 8:**
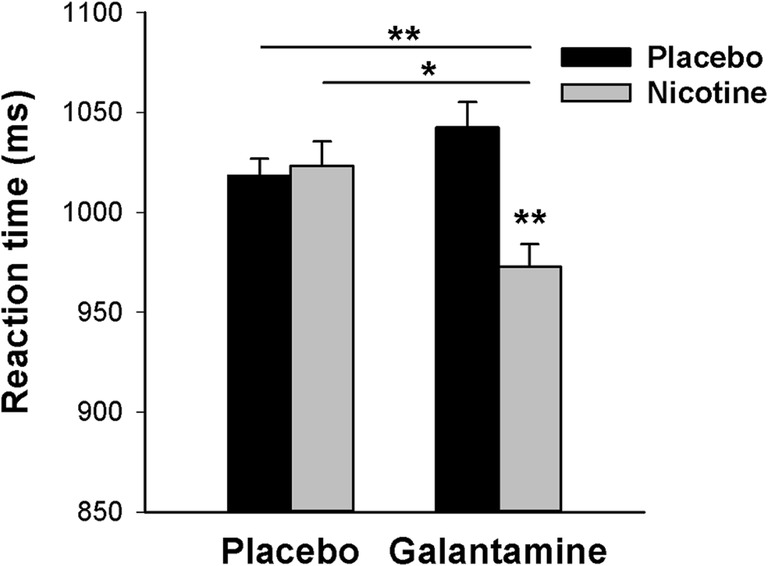
Effects of nicotine and galantamine on reaction time in the Change Detection Task, averaged over set sizes 1 and 5. Bars reflect the mean performance in each drug condition. Error bars reflect SEMs, adjusted to remove between-subject variability in the average performance across dose levels (Cousineau 2007; Morey 2008). *P < 0.05, **P < 0.01 in paired *t* tests

Responses were less accurate and slower for set size 5 than set size 1, as supported by significant main effects of set size for accuracy [*F*(1,25) = 286.7, *P* < 0.001, *η*_*p*_^2^ = 0.92] and RT [*F*(1,25) = 85.8, *P*s < 0.001, *η*_*p*_^2^ = 0.77]. For accuracy, no other main effects or interactions were significant. However, there was a significant nicotine × galantamine interaction on RT [*F*(1,25) = 8.40, *P* < 0.008, *η*_*p*_^2^ = 0.25], which remained significant even after Bonferroni correction for six analyzed performance measures. Figure 8 illustrates that the combined administration of nicotine and galantamine reduced RT relative to all other conditions, while the other conditions did not differ from each other. Thus, while neither nicotine nor galantamine alone had any effects by themselves, their combination acted synergistically to speed responding. This interaction did not depend on set size [nicotine × galantamine × set size interaction: *F*(1,25) = 0.02, *P* = 0.90, *η*_*p*_^2^ = 0.00].

To test whether RT reduction by the combined administration of nicotine and galantamine was related to AChE inhibition, RT and AChE activity values in the placebo session were subtracted from values in the nicotine + galantamine session, and the difference values underwent Pearson’s correlation. There was no significant correlation (*R* = − 0.29, *P* = 0.15), and the direction of the trend would be consistent with *less* AChE inhibition being associated with greater RT reduction.

## Discussion

The purpose of the present proof-of-concept study was to test whether a nAChR PAM could in principle augment the performance-enhancing effects of a nAChR agonist, employing nicotine and galantamine as pharmacological probes. Nicotine is a non-selective nAChR agonist, and similarly, no subtype selectivity of galantamine’s nAChR PAM action has been shown (Dajas-Bailador et al. 2003; Samochocki et al. 2003; Santos et al. 2002). A broad array of cognitive functions was probed, including stimulus detection under conditions of narrow focusing (SARAT predictive cue trials) and broad monitoring (non-predictive cue trials); rapid information processing, sustained attention, and working memory (RVIPT); and short-term memory (CDT). It was only on response time in the context of the CDT (a task in which response speed is not emphasized) that synergistic effects of nicotine and galantamine were observed, specifically, a reduction in RT by both drugs combined, but no effect of either drug alone.

A possible explanation for why only one of six performance measures showed the hypothesized interaction is that cognitive functions differ in their performance-optimal tone at nAChRs, or critical subtypes thereof. This would suggest that optimal doses of nAChR agonists and/or PAMs depend on the specific therapeutic effect desired. Performance of both SARAT and RVIPT improved with nicotine, and galantamine did not significantly increase these benefits, suggesting that nAChR tone at the critical subtypes may have been close to optimal for these attention-demanding tasks after nicotine administration. Thus, it is possible that galantamine would have potentiated effects of an even lower dose of nicotine on these measures; further research could test this possibility. In contrast, performance of the CDT was insensitive to nicotine alone, and RT benefits were seen only with the addition of galantamine. This suggests that cognitive functions reflected by this measure benefit from only a large increase in nAChR tone, and/or involve specific nAChR subtypes that either do not respond to concentrations of nicotine achieved here or are prone to desensitization by nicotine and benefited from a reversal of desensitization by galantamine’s PAM action.

The lack of effect of nicotine alone in the CDT which, contrary to SARAT and RVIPT, does not pose any significant challenge on attentional functions is consistent with the view that nicotine’s performance-enhancing effects are most robust in paradigms of attention (Hahn 2015; Newhouse et al. 2004; Stolerman et al. 1995). The present synergistic action with galantamine suggests that this lack of effect of nicotine alone may reflect an inability to achieve the right tone at the right nAChR subtype(s), rather than a general lack of nAChR involvement or reticence to nAChR-based modulation. The specific neuronal systems and nAChR subtypes that may be differentially involved in the cognitive functions probed by the CDT could only be speculated upon at this time. Data to date indicate that both α7 and α4β2* nAChRs, the most abundantly expressed nAChR subtypes throughout the brain (Gotti et al. 2009), mediate effects of nAChR agonists on attentional functions (e.g., Grottick et al. 2003; Hahn et al. 2011; Haydar and Dunlop 2010; Howe et al. 2010) and short-term memory (Levin et al. 2002; Rushforth et al. 2010). However, these functions may still be differentially influenced by a change in tone at these and other nAChR subtypes.

An alternative explanation for why only one of six performance measures showed the hypothesized interaction would be that additional pharmacological actions of galantamine confounded the results. A trend suggested that galantamine shortened SARAT RT independent of the presence or absence of nicotine, and correlation analysis indicated that this reflected residual AChE inhibition. Furthermore, galantamine alleviated the RVIPT performance decrement over time independent of the presence or absence of nicotine, consistent with a previous study testing a larger dose of galantamine (8 mg), whose effects presumably were dominated by AChE inhibition (Sofuoglu et al. 2012). An even smaller dose of galantamine may have achieved better separation of AChE inhibition from nAChR PAM effects. In this regard, it is of interest that the hypothesized nicotine × galantamine interaction was seen only in the task that was administered last, when galantamine blood levels were past their peak (Zhao et al. 2002). Clearly, a pure nAChR PAM would be a preferential tool to demonstrate positive allosteric potentiation of nAChR agonist effects, but no such compound is commercially available to date.

Despite galantamine’s additional mechanism(s) of actions, the synergistic effects with nicotine on CDT RT are informative. First, the nature of the interaction is conceptually explainable by galantamine’s PAM action facilitating nicotine effects, but not by AChE inhibition because a greater concentration of acetylcholine in the synaptic cleft would compete with nicotine for the same binding sites. This would reduce, not enhance, effects of nicotine, as seen with an 8-mg dose of galantamine (Sofuoglu et al. 2012). Second, the effect was not associated with AChE inhibition by galantamine as measured in blood, with even a trend association in the opposite direction. Thus, this finding strongly suggests that positive allosteric potentiation of cognitive-enhancing nAChR agonist effects is possible in principle.

While the present study was a proof of principle performed in healthy individuals, the ultimate target populations would be those with nAChR hypofunction such as people with schizophrenia, mild cognitive impairment, or Alzheimer’s disease, for which the nAChR agonist-PAM combination may be particularly beneficial. Combining a low-dose nAChR agonist and a PAM may represent a nAChR modulation strategy that is more fine-tuned and more sparing of native circuit dynamics than larger doses of agonist alone. nAChR subtype selectivity can be achieved with both nAChR agonists and PAMs; thus, a combination approach may achieve greater flexibility when targeting a critical subset of nAChR subtypes. For example, sub-threshold doses of a nAChR agonist selective for one group of nAChR subtypes may be combined with sub-threshold doses of a PAM selective for another group but overlapping with the first group on the critical subtype(s). Thus, the targeted co-administration of a nAChR PAM may enable the use of very small doses of nAChR agonist and achieve a narrower effects profile.

Limitations of the present study include a moderate sample size, a lack of verification of treatment blind fidelity, and the use of a single dose of both nicotine and galantamine. While additional test sessions would have made participant retention challenging, inclusion of multiple (and even smaller) doses of galantamine may have achieved better separation of AChE inhibition from nAChR PAM effects. Furthermore, given the absence of an in vivo marker of galantamine’s nAChR PAM action, we attributed its observed potentiation of nicotine effects to positive allosteric modulation based on the absence of an association with AChE inhibition. Given that AChE inhibition and nAChR positive allosteric modulation to date are recognized as the primary mechanisms of action of galantamine, positive allosteric modulation would appear a likely mediator of the effects of galantamine reported here, but our inability to measure it directly is a limitation. Finally, neither nicotine’s agonist effects nor galantamine’s PAM effects are selective for any specific subtypes of the nAChR. Thus, the present findings provide a proof of principle, but do not advance the more targeted strategies outlined in the previous paragraph.

In summary, the present findings suggest that positive allosteric potentiation of cognitive effects of exogenous nAChR agonists is possible in principle and encourage further study of this mechanism with even smaller doses of nAChR agonist and with novel nAChR PAMs devoid of additional pharmacological actions.
